# Comparative safety assessment of genetically modified crops: focus on equivalence with reference varieties could contribute to more efficient and effective field trials

**DOI:** 10.1007/s11248-023-00344-y

**Published:** 2023-05-22

**Authors:** Gijs A. Kleter, Hilko van der Voet, Jasper Engel, Jan-Pieter van der Berg

**Affiliations:** 1 Wageningen Food Safety Research, Part of Wageningen University and Research, P.O. Box 230, 6700 AE Wageningen, Netherlands; 2 Biometris, Wageningen Plant Research, Part of Wageningen University and Research, Wageningen, Netherlands

**Keywords:** Genetically modified crops, Comparative safety assessment, Field trials, Crop composition, Statistical analysis, Equivalence testing

## Abstract

The initial compositional analysis of plants plays an important role within the internationally harmonized comparative safety assessment approach for genetically modified plants. Current EFSA guidance prescribes two types of comparison, namely difference tests with regard to a conventional comparator or control, and equivalence tests with regard to a collection of commercial reference varieties. The experience gained so far shows that most of the statistically significant differences between the test and control can be discounted based on the fact that they are still within equivalence limits of reference varieties with a presumed history of safe use. Inclusion of a test variety and reference varieties into field trial design, and of the statistical equivalence test would already suffice for the purpose of finding relevant parameters that warrant further assessment, hence both the inclusion of a conventional counterpart and the performance of difference testing can be omitted. This would also allow for the inclusion of safety testing regimes into plant variety testing VCU (value for cultivation and use) or other, independent variety trials.

## Introduction

The first large-scale commercialization of genetically modified (GM) crops obtained through recombinant DNA technology took place in the mid-1990s. Since then, the adoption of this technology by farmers worldwide has steadily increased, reaching 190 million hectares in the year 2019. Most (91%) of this acreage was located in the USA, Brazil, Argentina, Canada, and India. A major share (99%) of these crops were commodity crops, in particular soybean (48%), as well as maize, cotton, and oilseed rape. The most prevalent types of traits that have been introduced into these crops are herbicide tolerance and insect resistance (ISAAA 2019).

Before GM crops can be marketed, they usually undergo a pre-market regulatory approval procedure, which includes an assessment of the safety of the product for human and animal health and the environment. A key feature of the safety assessment is the identification of potential changes in the GM crop caused by the genetic modification that might cause concern over its safety. Towards this end, the new crop is compared with its non-modified counterpart with a history of safe use and consumption.

As biotechnology continues to advance, the question remains whether the regulatory approval procedures and the cognate safety assessment requirements for recombinant DNA organisms are able to keep up or should be adjusted. For example, gene editing techniques are claimed to have lowered the threshold for the use of genetic tools. They reportedly afford greater precision than more traditional forms of genetic modification such as recombinant DNA-based genetic modification and mutagenesis involving the use of mutagenic chemicals and physical agents that cause permanent changes to the host’s DNA, such as ethyl methanesulfonate and ionizing radiation, respectively. Yet the products of gene editing are currently still treated in the same way as other GM products by some but not all regulators worldwide. A common reason for not treating them in the same way is that certain small mutations caused by gene editing methods are indiscernible from both natural ones and those caused by conventional breeding or mutation breeding. With gene editing now within reach of small and medium-sized enterprises and academics, it is pivotal to ensure that safety testing should not unduly impede innovation but be risk-proportionate and commensurate with measures for other products with similar risk profiles.

This paper proposes a potential improvement to the current set of comparative experiments with new, genetically improved varieties of crops (e.g., test vs. control; test vs. references) by whittling them down to the most meaningful comparison of test-*versus-*references. Moreover, it recommends accommodating these studies into the variety registration field trials for cultivation and agronomic value.

### The comparative safety assessment approach

The food, feed, and environmental safety assessment of GM crops follows an internationally harmonized comparative approach. There have been historic efforts of various international organizations, such as the Food and Agriculture Organization of the United Nations (FAO), World Health Organization (WHO), and Organization for Economic Co-operation and Development (OECD), towards international harmonization of this comparative risk assessment approach. This started already in the 1980s, a long time before the first GM crops entered the market. These efforts culminated, for instance, in the publication of the Codex Alimentarius guidelines for the risk assessment of foods derived from crops obtained through recombinant DNA technology, in 2003 (Codex Alimentarius 2008).

In the Codex Alimentarius guidelines, the concept of “substantial equivalence” is an initial key step in the comparative safety assessment. It is neither a safety assessment, nor does it imply absolute safety of a new food; it rather is a starting point with the aim of structuring the assessment of a new food. Originally, it was based on a comparison of the new food with a “conventional counterpart” having a history of safe use (as described below, later also comparisons with other varieties of the species were considered, to allow for accepted variations). The differences (both intended and unintended) and similarities thus found should be assessed for their biological significance based on, for example, the range of natural variation. This helps to identify possible issues for the safety and nutritional value of the new food. The reason for this is that foods from many crop plant species are complex mixtures of a range of compounds with potentially beneficial and adverse effects. Instead of attempting to find all hazards within the new plant-derived food, the comparative safety assessment focuses on identifying those hazards that are new or changed with respect to the conventional counterpart (Codex Alimentarius 2008).

The comparative safety assessment of GM plants follows a stepwise process, focusing on a number of relevant items: the description of the genetically modified plant, including the host plant and its history of food use; the donor organism(s) of the recombinant DNA; the genetic modification (such as the procedure, the introduced DNA and its function); the trait(s) introduced; the safety assessment, considering both intended and unintended effects, for example any newly expressed non-nucleic-acid substances, such as proteins (e.g., toxicity, allergenicity); changes found during the compositional analysis of key substances, and any metabolites formed; the impact of processing thereon and their nutritional implications; and any other considerations (Codex Alimentarius 2008).

The compositional analysis of key components in samples obtained from field trials to be performed with the new plant variety and its comparators (both the conventional counterpart and additional reference varieties) plays an important role in this assessment, particularly for the identification of unintended effects. This adds to the analysis of agronomic and phenotypic characteristics already carried out by breeders when selecting new varieties for marketing. All these analyses will help ensure that substances that are relevant for either safety or nutrition have not been changed in such a way that this could have adverse effects on consumers’ health (Codex Alimentarius 2008).

For the comparison, both the GM plant and its conventional counterpart should be grown under the same conditions. The counterpart should ideally be near-isogenic, whilst it has previously been observed that the genetic relationship between GM crop and its comparator may vary in practice, depending on the breeding schemes followed for both, as well as the complexity of the GM crop, such as in the case of “stacked” GM traits (EFSA 2011c). This “as close as possible” genetic similarity between GM crop and its control helps avoiding the introduction of additional dissimilarities that would interfere with the identification of unintended differences caused by the genetic modification per se (EFSA 2011c; NRC 2004). In addition, the trial locations should be representative of the environments where the GM plant variety is to be grown commercially. The number of trial sites should allow to accurately establish the plants’ compositional characteristics across this range of environments. In addition, trial locations should be replicated so as to reduce the possible impacts of local environmental and genotypic variability (Codex Alimentarius 2008). More detailed guidelines with respect to field trial design and the statistical analysis are provided, for instance, by Annex II to the European Implementation Regulation (EU) 503/2013 (EC 2013).

As for the key crop compositional components to be analysed, the OECD Working Party for the Safety of Novel Foods and Feeds has published a series of consensus documents for a wide range of food and feed crops, including alfalfa, apple, barley, cassava, common bean, cotton, cowpea, grain sorghum, low-erucic-acid rapeseed (canola), maize, papaya, potato, rice, soybean, sugar beet, sugarcane, sunflower, sweet potato, tomato, and wheat (OECD, website). Table 1, as an example, lists the constituents that the OECD consensus document for potato tubers recommends for analysis (OECD 2021).

**Table 1:** Nutritional and compositional parameters of potato tuber suggested for analysis for food and feed uses (OECD 2021)

Constituent	Food	Feed
Moisture	X	X
Protein	X	X
Fat	X	X
Carbohydrates	X	X
Ash	X	X
Dietary fibre	X	
Fibre (ADF, NDF)*		X
Starch	X	
Vitamin C (ascorbic acid)	X	
Vitamin B6 (pyridoxin)	X	
Potassium	X	
Magnesium	X	
Total glycoalkaloids	X	X

^*^ADF, Acid detergent fibre; NDF, neutral detergent fibre

### Difference and equivalence testing: the current EU approach

Whilst the Codex Alimentarius guidelines have found worldwide application, various of its elements such as the comparative analysis have been elaborated into greater depth by the European Union (EU). This is exemplified by the guidance annexed to Implementing Regulation (EU) No 503/2013. An outstanding feature is the statistical approach which it lays out for the comparative analysis. The groundwork which has led to these recommendations was published by EFSA and the experts it had convened for this (EFSA 2011a; van der Voet et al. 2011). The EFSA statistical methodology applied the concept of equivalence testing besides that of difference testing. In the case of difference testing, the null-hypothesis is that plant characteristics are the same and that evidence is needed to prove that they are actually different. With a significance level of 0.05, for example, the error of falsely concluding that a mean plant characteristic is different when it is actually the same is limited to 5%, whilst that of correctly concluding that there is a difference of a specified magnitude constitutes the statistical power. With equivalence testing, the null hypothesis is that there is a difference of at least a certain size, and rejecting this null hypothesis constitutes a proof of equivalence. This approach controls the error of falsely concluding equivalence between the test and references at a specific level, for example 5%. In the EFSA/EU approach, the equivalence of the new crop to non-GM reference varieties with a history of safe use is at the focus of the comparative assessment.

Before this approach was introduced, difference testing had been performed in most of the safety dossiers provided to the EFSA GMO Panel, identifying statistically significant differences whereas equivalence testing was seldom performed. As a complement to the difference test and a proxy for the equivalence test, value ranges (minimum–maximum) or confidence intervals of reference varieties tested in the same or other field trials were used. This way, the differences found were offset against the background variability in commercial varieties. The new methodology introduced by EFSA and the EU legislation thus constitutes a homogenisation, consolidation, and refinement according to statistically sound principles regarding the choice of null hypothesis and the type of error that should be controlled.

**Fig. 1:**
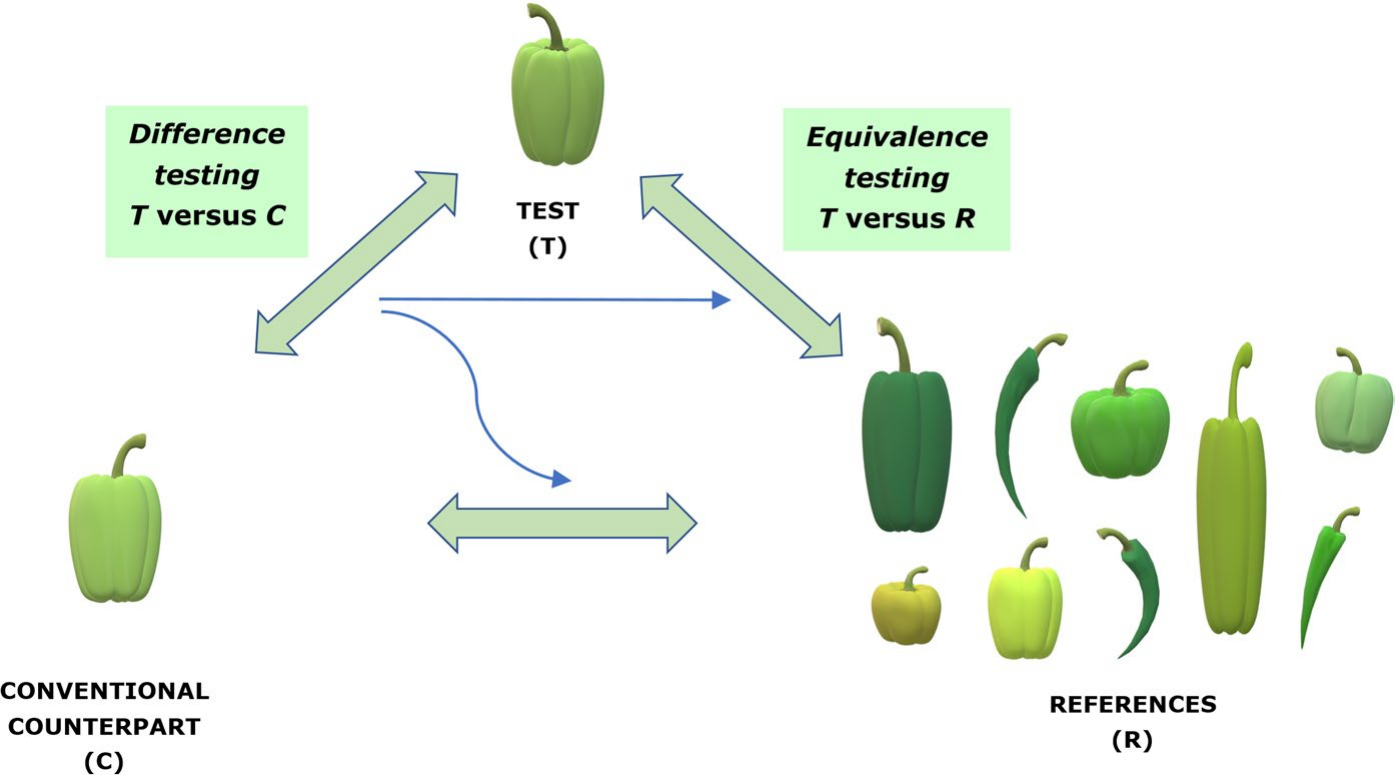
The various comparisons between test, control, and reference varieties (with *Capsicum* varieties as a hypothetical example)

When focusing on the role of the conventional counterpart and the non-GM commercial reference varieties with a history of safe use, the EFSA/EU approach is to combine a traditional test for differences (T-*versus*-C difference test) between T (Test genotype, GM crop) and C (Conventional counterpart) with an equivalence test to show that T is within the bounds of a set of R (Reference) varieties (T-*versus*-R equivalence test) (Fig. 1).

**Fig. 2:**
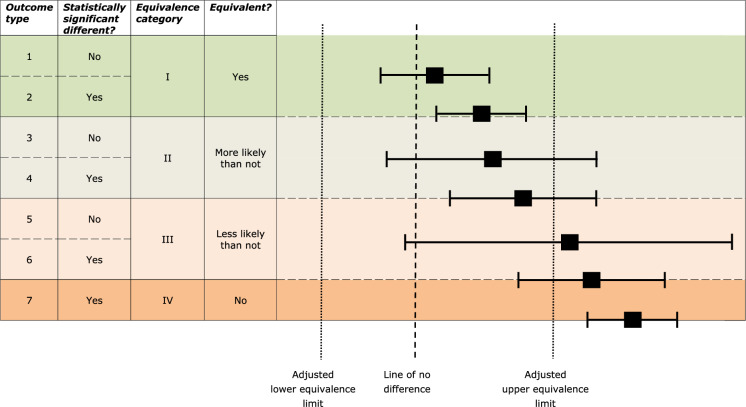
Graphical display for observed differences, difference and equivalence tests (modified from EU 2013). Shown are: (horizontal lines) the confidence interval (CI) for the difference, and (vertical lines) lower and upper equivalence limits and the line of no difference. A significant difference is shown when the line of no difference is outside the CI. Equivalence is shown when the CI falls completely inside the lower and upper equivalence limit

The two tests are complementary: the T-*versus-*C difference test identifies biological changes that may require further investigation on a case-by-case basis; the T-*versus-*R equivalence test gives confidence that the mean compositional characteristics per analyte of T are within the range of those of the commercial R varieties, whether or not there is a significant difference from the control variety. In Implementing Regulation (EU) No 503/2013, less weight is put on binary test results, and more weight on the broader assessment of quantitative differences between T, C and R varieties (EU 2013) (Fig. 2).

In addition, the annex to Implementing Regulation (EU) No 503/2013 as well as a document published by a dedicated EFSA GMO Panel Working Group in 2011 also provide guidance for the requirements of the conventional counterpart and the additional commercial reference varieties to be included in the field trials, (EFSA 2011c; EU 2013).

The choice of the conventional counterpart needs to be explained by the applicant, including information on the genetic distance and a breeding pedigree. For single GM events in a vegetatively propagated host crop, the conventional counterpart should be the isogenic non-GM line, whilst for sexually propagated ones, this should be a near-isogenic line, which is genetically identical to the GM plant except for some loci. A negative segregant, i.e., non-GM progeny of hemizygous GM plants obtained through the same transformation protocol, will not be accepted as a conventional counterpart, but may be included as an additional comparator. When there is no appropriate conventional counterpart with a history of safe use available, or if the modification is such that the conventional counterpart cannot be identified, the safety assessment will need to be performed as if the new food or feed product is a Novel Food, under Regulation (EU) No 2015/2283 (EFSA 2011c; EU 2013). Notably, for maize and cotton, stacked GM varieties are nowadays the norm. Under EU regulations, the regulatory application for a stacked event from previously approved single events has to be filed separately, whilst the assessment will then focus on the potential occurrence of interactions between the events that could affect safety. A conventional counterpart may not always be available for the comparative analysis of *stacked* events, though. This potential scenario has also been acknowledged, in which case other appropriate counterparts should be sought (EFSA 2011c; EU 2013).

With regard to the reference varieties, the Implementing Regulation (EU) No 503/2013 requires the inclusion of at least six but preferably more non-GM reference varieties with a history of safe use, to characterise usual variation for the purpose of equivalence testing. These reference varieties should be appropriate for the test locations, and this should be justified explicitly. The minimum number of trial sites for the comparative assessment of compositional, agronomic, and phenotypic traits is eight. As for the number of reference varieties, this number of trial sites has been set based on pragmatic considerations, providing a basis for environmental variability representative of commercial production that is also sufficient for gauging the variability between test materials across different environments (EFSA 2010; EU 2013).

### Comparators and follow-up to equivalence testing: the European experience

An overview of comparators as described in EFSA GMO Panel Opinions on single and stacked events in the past 5 years (2017–2021) is provided in Table 2. We did not consider earlier opinions because only from the end of 2016 on, detailed descriptions of the basis for the similarity between test, conventional counterpart, and references were given. From these Opinions, which form the basis for the European decisions on market approval applications, it may be observed that the description of the genetics of the comparator commonly includes terms such as “near-isogenic”, “similar”, “highly similar”, and “same”. Frequently the pedigree or the breeding procedure starting from the initial transformation is also cited in support of such conclusions. For example, the same inbred parental varieties (e.g., LH244, LH287) as used for creating GM maize hybrids but without the GM events have been crossed to create the conventional counterpart. The opinions, though, do not provide specific information on the history of commercial use of these comparators, for which reason it is unclear to what extent this has been considered by the Panel. This information therefore had to be searched elsewhere, such as in US plant patents. It thus showed that most of the germplasm listed as comparator genetic background in Table 2 had a record of use or registration as cultivar or breeding line, whilst this remained unclear for 3 maize, 1 oilseed rape, and 1 soybean comparators. For the methodological issues in this paper, we therefore assumed that the selected comparators do have a history of safe use. The EFSA GMO Panel has previously noted that the concept of history of safe use has not been developed for plant breeding per se but for the safety of imported foods. It also noted that for certain crops, varieties may have a short life cycle (e.g., 2–3 years for some), such as for wheat, potato, and maize. It therefore concluded that for such crops, the history of safe use does not pertain to specific varieties or genes but more generally to the plant species (EFSA 2012).

**Table 2:** Genetic background and history of application of non-GM conventional counterparts used in the field trials for compositional analysis for applications for single and stacked events in EFSA GMO Panel opinions published in 2017–2021*

Event	Genetic background	Cultivar/breeding line?	Comment	Reference
Cotton				
*Single events*				
GHB811	Coker 312	Yes	Old variety	EFSA (2021a), National Cotton Council (1997)
MON 88701	Coker 130	Yes	No longer sold	EFSA (2017a), Texas A&M (2001)
*Stacked events*				
GHB614 × LLCotton25 × MON 15985	FiberMax958 (FM958)	Yes	Introduced in 2000	EFSA (2018a), Dever (2000)
GHB614 × T304‐40 × GHB119	Coker 312	Yes	Old variety	EFSA (2018b), National Cotton Council (1997)
Maize				
*Single events*				
4114	PH705 × PHW2Z; PH12SG × PHW2Z	Yes	Hybrid	EFSA (2018c), Hoffbeck (2005), Smalley (2011), Verde Chifflet (2011)
DAS-40278-9	XHH13 × 7SH382	Yes	Hybrid	EFSA (2016a), Johnson (2002), Johnson (2010)
MON 87403	LH244 × LH287	Yes	Hybrid	EFSA (2018d), Armstrong (2001), Foley (2001)
MON87411	LH244 × HCL645	Yes / ?	Hybrid; HCL645 = company accession	EFSA (2018e), Armstrong (2001), Wu et al. (2019)
MZHG0JG	NP2222 × NP2391	Yes	Hybrid	EFSA (2018f), Delzer (2004, 2007)
MZIR098	NP2391 × NP2222	Yes	Hybrid	EFSA (2020d), Delzer (2004, 2007)
*Stacked events*				
1507 × 59122 × MON810 × NK603	PH09B × PH581	Yes	Hybrid	EFSA (2017h), Carlone and Noble (2004), Williams (1999)
1507 × MIR162 × MON810 × NK603	PHE4N × PHH9H	Yes	Hybrid	EFSA (2021b), Fox and McIntosh (2009), Fox (2009)
Bt11 × MIR162 × MIR604 × 1507 × 5307 × GA21	5XH751 × NP2222	?/Yes	Hybrid; 5XH751 = company accession	EFSA (2019a, p. 80) of Cai et al. (2020), Delzer (2004)
Bt11 × MIR162 × 1507 × GA21	5XH751 × NP2222	?/Yes	Hybrid; 5XH751 = company accession	EFSA (2018g, p. 80) of Cai et al. (2020), Delzer (2004)
MON 87427 × MON 87460 × MON 89034 × 1507 × MON 87411 × 59122	LH244 × LH287	Yes	Hybrid	EFSA (2021c), Armstrong (2001), Foley (2001)
MON 87427 × MON 87460 × MON 89034 × MIR162 × NK603	LH244 × LH287	Yes	Hybrid	EFSA (2019b), Armstrong (2001), Foley (2001)
MON 87427 × MON 89034 × 1507 × MON 88017 × 59122	HCL301 × LH287	Yes	Hybrid	EFSA (2017b, p. 16) of SNICS (2014), Foley (2001)
MON 87427 × MON 89034 × MIR162 × MON 87411	LH244 × LH287	Yes	Hybrid	EFSA (2019c), Armstrong (2001), Foley (2001)
MON 87427 × MON 89034 × MIR162 × NK603	LH244 × LH287	Yes	Hybrid	EFSA (2019d), Armstrong (2001), Foley (2001)
MON 87427 × MON 89034 × NK603	LH198 × LH287	Yes	Hybrid	EFSA (2017c), Miller (1994), Foley (2001)
MON 89034 × 1507 × MON 88017 × 59122 × DAS‐40278‐9	SLB01 × BE9514	Yes	Hybrid	EFSA (2019e), Bohning (2007), Bohning (2009)
MON 89034 × 1507 × NK603 × DAS‐40278‐9	7SH382 × XHH13	Yes	Hybrid	EFSA (2019f), Johnson (2002), Johnson (2010)
NK603 × T25 × DAS-40278–9	SLB01 × PHRDW	Yes	Hybrid	EFSA (2021d), Bohning (2009), Piper and Hotchkiss (2010)
Oilseed rape				
*Single events*				
73496	5536 F × 5676 M	?/?	Hybrid; 5536 F = company accession; 5676 M bred specifically for this comparison	EFSA (2021e, p. 13) of Pioneer Hi-Bred International (2011, p. 21) of HCB (2013)
*Stacked events*				
MON 88302 × MS8 × RF3	Ebony	Yes		EFSA (2017d), CFIA (1995)
Soybean				
*Single events*				
DAS-44406-6	Maverick	Yes		EFSA (2017e), Sleper et al. (1998)
DAS-68416-4	Maverick	Yes		EFSA (2017f), Sleper et al. (1998)
DAS-81419-2	Maverick	Yes		EFSA (2016b), Sleper et al. (1998)
GMB151	Thorne	Yes		EFSA (2021f), McBlain et al. (1993)
MON 87751	A3555	Yes		EFSA (2018h), Asgrow (2019)
SYHT0H2	Jack	Yes		EFSA (2020a), Nickell et al. (1990)
*Stacked events*				
DAS-81419-2 × DAS-44406-6	Maverick	Yes		EFSA (2020c), Sleper et al. (1998)
FG72 × A5547-127	MST24 and MST39	?/?	MST = MS Technology (company)	EFSA (2017g)
MON 87705 × MON 87708 × MON 89788	A3525	Yes		EFSA (2020b), Seedquest (2003)
MON 87708 × MON 89788 × A5547‐127	A3555	Yes		EFSA (2019g), Asgrow (2019)
MON 87751 × MON 87701 × MON 87708 × MON 89788	A3555	Yes		EFSA (2019h), Asgrow (2019)

*Renewal applications not included

Notably, many of these recent EFSA opinions mention the maturity groups in support of the comparability and appropriateness of the test, conventional counterpart, and reference varieties (Table 3). Maturity ratings indicate to which latitudes with a particular photoperiod and temperature the particular variety is adapted and will perform optimally. From Table 3 it follows, that test, control, and reference materials were similar within the particular field trials.

**Table 3:** Maturity classes of test and conventional counterpart varieties, and references of maize and soybean varieties for which these were provided in EFSA GMO Panel opinions on single and stacked events in 2017–2021

Event	Genetic background of test and control	Number of reference varieties	Maturity, test and control	Maturity, references	Reference
Maize					
*Single events*					
MON87403	LH244 × LH287	17	110	109–115	EFSA (2018d)
MZHG0JG	NP2222 × NP2391	6	105–107	93–115	EFSA (2018f)
MZIR098	NP2391 × NP2222	6	105–107	93–115	EFSA (2020d)
*Stacked events*					
1507 × MIR162 × MON810 × NK603	PHE4N × PHH9H	14	107	104–111	EFSA (2021b)
MON87427 × MON87460 × MON89034 × 1507 × MON87411 × 59122	LH244 × LH287	18	110	108–115	EFSA (2021c)
MON87427 × MON87460 × MON89034 × MIR162 × NK603	LH244 × LH287	18	110	108–115	EFSA (2019b)
MON87427 × MON89034 × MIR162 × MON87411	LH244 × LH287	20	112	107–115	EFSA (2019c)
MON87427 × MON89034 × MIR162 × NK603	LH244 × LH287	17	110	109–115	EFSA (2019d)
Soybean					
*Single events*					
GMB151	Thorne	9	3	2.2–3.4	EFSA (2021f)
*Stacked events*					
FG72 × A5547-127	MST24 and MST39	6	2 and 3	2 and 3	EFSA (2017g)
MON87705 × MON87708 × MON89788	A3525	18	3.5	2.8–4.2	EFSA (2020b)
MON87708 × MON89788 × A5547‐127	A3555	16	3.5	3.0–3.9	EFSA (2019g)
MON87751 × MON87701 × MON87708 × MON89788	A3525, A3555	18	3.5	2.8–4.1	EFSA (2019h)

With regard to the outcomes of equivalence testing performed in most of the application studies on which the Panel gave its opinion in the period 2017–2021, the Panel followed a tiered approach, as follows:

1. Difference and equivalence testing, in parallel:
  - Statistically significant differences between test and conventional counterpart are identified T versus C, Fig. 1). For herbicide-tolerant crops, there are commonly multiple test groups, namely test plants treated with the target herbicide(s) and another group of test plants treated with conventional herbicides. This may also account for the relatively high number of differences in some cases, where up to five different test groups were tested in parallel.
  - Parameters falling within Equivalence categories III (non-equivalence more likely than not) and IV (non-equivalence) (T versus R, Fig. 1).
2. Identify those parameters which both are statistically significantly different (between test and control) and fall within the equivalence categories III and IV (or for which no equivalence limits could be established). Table 4 shows that despite large numbers of statistically significant differences found in a number of cases, only a fraction of these fall within equivalence categories III and IV. In many instances, they make up most of the not-shown equivalences falling into these categories. In these cases, the remainder within equivalence categories III/IV consist of no or only a few parameters with a not-shown equivalence between the GM crop and the reference varieties but without a statistically significant difference between the GM crop and the conventional counterpart. Hence the number of parameters identified in this stage usually is far below the total number of differences and slightly below that of all not-shown equivalences.
3. Assess the parameters showing both differences and not-shown equivalences further for potential toxicity and allergenicity, and for nutritional implications. The potential safety implications of the changes in these parameters are commonly summarized in the respective sections of the opinions dealing with potential toxicity, allergenicity, and nutritional impact. In the opinions screened for this study (Table 4), the Panel often concluded that the changes did not raise concerns considering the magnitude of the difference and the known biological function of the implicated parameters.

**Table 4:** Number of statistically significant differences ($\alpha =0.05$) and parameters not showing equivalence (categories III/IV) identified for compositional parameters in GM crops’ edible parts in opinions of the EFSA GMO Panel (2017–2021) for events for which the equivalence test was performed

Event	Number of reference varieties	Number of endpoints (total/tested)*	Statistical findings				Reference
			Number of comparisons	1. Statistically significant differences	2 Parameters not-showing equivalence (cat. III/IV)	Both 1 and 2	
Cotton							
*Single events*							
GHB811	7	73/56	112	29	2	1	EFSA (2021a)
GHB614 × T304‐40 × GHB119	6	73/53	106	68	8	6	EFSA (2018b)
Maize							
*Single events*							
4114	19	84/71	142	68	0	0	EFSA (2018c)
DAS-40278-9	6	82/59	236	80	24	20	EFSA (2016a)
MON87403	17	78/62	62	58	2	0	EFSA (2018d)
MON87411	22	78/62	124	50	2	1	EFSA (2018e)
MZHG0JG	6	82/66	132	63	3	0	EFSA (2018f)
MZIR098	6	81/66	132	23	4	1	EFSA (2020d)
*Stacked events*							
1507 × MIR162 × MON810 × NK603	14	84/71	142	80	0	0	EFSA (2021b)
Bt11 × MIR162 × 1507 × GA21	6	65/59	118	8	6	6	EFSA (2018g)
Bt11 × MIR162 × MIR604 × 1507 × 5307 × GA21	6	82/64	128	49	24	14	EFSA (2019a)
MON 87427 × MON 87460 × MON 89034 × 1507 × MON 87411 × 59122	18	78/63	126	69	9	7	EFSA (2021c)
MON 87427 × MON 87460 × MON 89034 × MIR162 × NK603	18	78/63	126	85	1	0	EFSA (2019b)
MON 87427 × MON 89034 × 1507 × MON 88017 × 59122	24	78/62	124	97	1	1	EFSA (2017b)
MON 87427 × MON 89034 × MIR162 × MON 87411	20	78/63	126	82	1	1	EFSA (2019c)
MON 87427 × MON 89034 × MIR162 × NK603	17	78/63	126	58	1	0	EFSA (2019d)
MON 87427 × MON 89034 × NK603	22	79/65	130	58	3	3	EFSA (2017c)
MON 89034 × 1507 × MON 88017 × 59122 × DAS‐40278‐9	6	82/65	130	57	17	11	EFSA (2019e)
MON 89034 × 1507 × NK603 × DAS‐40278‐9	6	82/65	130	52	18	10	EFSA (2019f)
NK603 × T25 × DAS-40278-9	20	81/72	144	60	2	0	EFSA (2021d)
Oilseed rape							
*Single events*							
73496	6	131/103	206	109	11	7	EFSA (2021e)
*Stacked events*							
MON 88302 × MS8 × RF3	15	71/59	118	41	0	0	EFSA (2017d)
Soybean							
*Single events*							
DAS-44406-6	11	87/67	335	115	n.a	16	EFSA (2017e)
DAS-68416-4	6	87/64	256	91	n.a	19	EFSA (2017f)
DAS-81419-2	9	87/66	66	26		1	EFSA (2016b
GMB151	9	112/89	178	67	6	2	EFSA (2021f)
MON 87751	19	74/58	58	17	0	0	EFSA (2018h)
SYHT0H2	6	66/60	120	59	7	3	EFSA (2020a)
*Stacked events*							
DAS-81419-2 × DAS-44406–6	6	107/87	174	71	13	3	EFSA (2020c)
FG72 × A5547-127	6	82/61	122	67	0	0	EFSA (2017g)
MON 87705 × MON 87708 × MON 89788	18	74/60	120	68	17	17	EFSA (2020b)
MON 87708 × MON 89788 × A5547‐127	16	74/60	120	56	4	4	EFSA (2019g)
MON 87751 × MON 87701 × MON 87708 × MON 89788	18	74/60	120	42	1	1	EFSA (2019h)

*There are various reasons for a lower number of tested endpoints than the total one, including endpoints for which the majority of results were below the level of detection or quantification, data not being amenable to equivalence testing (e.g., too little variability), or dryness/moisture of harvested and stored seeds

n.a. = information not available in the cited reference

## Discussion

It has been a decade since the parallel difference and equivalence testing were recommended by respectively the EFSA GMO Panel and the Implementing Regulation (EU) 503/2013 for analysing compositional data of GM crops and their counterparts as key elements of a GMO safety assessment. With the knowledge and experience thus gained, it appears timely to gauge if possible adjustments could help increase the efficiency and effectiveness of the statistical data processing.

As seen in Table 4, the number of parameters where equivalence with the reference variety set was not shown (Equivalence categories III and IV) is much smaller than for the number of significant differences with the control. Apparently, values of many parameters that differ significantly from the control still fall within the background spanned by the reference varieties, according to the EFSA statistical procedure. Notably, only a minor part of all statistically significant differences falls within equivalence categories III and IV and would therefore warrant closer scrutiny (Table 4). In many cases, the number of parameters not-showing equivalence (category III and IV) was only slightly higher than those not showing equivalence *and* showing a statistically significant difference. This begs the question if equivalence testing per se might not already be sufficient for the purpose of selecting parameters that require further evaluation of their possible safety and nutritional implications.

Recent studies indicate that the equivalence testing approach per se is also amenable to improvement. The current European approach to equivalence testing (EFSA 2011b; EU 2013) is essentially a two-step approach, where equivalence limits are estimated in the first step, and the equivalence tests are performed in the second step. Since the uncertainty of the equivalence limit estimate is disregarded in the second step, the equivalence test is only approximate. Moreover, the method devised by EFSA affords only limited control of the statistical power.

Kang and Vahl (2014) proposed an alternative method that integrates all uncertainties by using a general statistical approach designated as generalised fiducial inference (e.g., Hannig et al. 2016). Vahl and Kang (2016) introduced the concept of distribution-wise equivalence (DWE) testing which proposes to compare distributions rather than mean values and takes also the within-variety between-plot variation into account. Building on this, the difference and equivalence testing approach was adapted to data from animal feeding studies in the G-TwYST project (van der Voet et al. 2017; Steinberg et al. 2019). In animal studies there cannot be sufficient reference varieties in each study. However, some reference varieties may have been included, and information may be pooled over a set of historical data. Therefore, an approach was developed where the T-C difference was compared to typical differences between reference varieties, i.e. differences between any pair of randomly chosen reference varieties (T-C approach to equivalence testing). The test was devised to guarantee a desired statistical power. Recently, a similar DWE desired power testing approach directly comparing test and references has also been adapted for compositional data sets (T-R equivalence test, see Engel and van der Voet 2021).

Omitting the conventional counterpart from the assessment would render the comparative assessment amenable to inclusion into variety registration trials, for which similar field trials need to be performed. Two main categories of variety registration trials can be discerned, namely the DUS and VCU trials. DUS stands for “distinct, uniform, and stable” and relates to variety specific traits that need to be distinct (e.g., in crop morphology, physiology, traits), uniformly transferred during propagation, and stable during repeated propagation. The nature and layout of such trials and parameters to be tested may be different from one crop to another. UPOV (International Union for the Protection of new Varieties of Plants) has established a wide range of guidelines for food, forestry, and other crops (UPOV, website).

More relevant for the comparative assessment are the Value-for-Cultivation-and-Use (VCU) trials. These focus on performance, compositional quality and, for example, stress and disease resistance. Protocols for VCU trials may differ from one country to another whilst general various reference lines need to be included, including general and newly recommended ones. In addition to this, also other, complementary independent field trials are performed by the seed industries in various countries. Locations should be representative of the various conditions that the crop will be grown under commercially, and trials site layout will include replications (e.g., Dutch Plant Variety Board 2017) This may include the use of reference varieties and may require field trials to be performed over multiple years or seasons.

Instead of demanding two types of pre-market field trials, it would be efficient to add equivalence testing of various relevant, safety-related compositional characteristics as an additional criterion to VCU variety testing protocols. It seems well-justified to have a closer look at the efficiency of, and indeed overlap in standing procedures in this respect (Slot et al. 2018). This should also include the use of data from reference varieties. Whether these data should be derived from the same field trials, or whether it is scientifically justified to use historical data as has already been compiled in databases, expanding the latter simultaneously along the way, may be further discussed. Notably, various recent reports studied the interface between biosafety and variety registration systems in different countries, particularly Sub-Saharan African nations, stressing the importance of harmonization of field testing requirements and avoidance of overlap, as is already practice in Kenya, for example (Akinbo et al. 2021; Anjanappa and Gruissem 2021; Komen and Wafula 2021). Moreover, authors from diverse backgrounds (governmental, corporate) in other jurisdictions take a step further by recommending the abandonment of the mandatory, process-based requirement for field trials testing compositional, agronomic, and phenotypic characteristics of GM crops in exchange for a case-by-case approach based on risk characteristics (Herman and Price 2013; Vesprini et al. 2022).

In conclusion, experience has accrued over the last 10 years with comparative field trials with GM crops performed according to EU guidelines for their design, execution, and statistical analysis (EU 2013). It shows that the statistical difference test and the conventional counterpart can be omitted without compromising on the final outcomes of this comparison whilst focusing on the equivalence between the GM crop and reference varieties. Such a streamlined setup would also be amenable to inclusion into VCU trials for variety registration, thereby further enhancing the effectiveness and efficiency of these field trials.
